# Highly Variable Dietary RNAi Sensitivity Among Coleoptera

**DOI:** 10.3389/fpls.2021.790816

**Published:** 2021-12-07

**Authors:** Jonathan Willow, Eve Veromann

**Affiliations:** ^1^Chair of Plant Health, Institute of Agricultural and Environmental Sciences, Estonian University of Life Sciences, Tartu, Estonia; ^2^Department of Plants and Crops, Laboratory of Agrozoology, Faculty of Bioscience Engineering, Ghent University, Ghent, Belgium

**Keywords:** RNA interference, dsRNA, plant biotechnology, beetle, biological variability, biopesticide, insecticide, dietary RNAi

## Abstract

Many herbivorous beetles (Order Coleoptera) contribute to serious losses in crop yields and forest trees, and plant biotechnology solutions are being developed with the hope of limiting these losses. Due to the unprecedented target-specificity of double-stranded RNA (dsRNA), and its utility in inducing RNA interference (RNAi) when consumed by target pest species, dsRNA-based plant biotechnology approaches represent the cutting edge of current pesticide research and development. We review dietary RNAi studies in coleopterans and discuss prospects and future directions regarding RNAi-based management of coleopteran plant pests. Herein, we also provide a balanced overview of existing studies in order to provide an accurate re-assessment of dietary RNAi sensitivity in coleopterans, despite the limitations to the existing body of scientific literature. We further discuss impediments to our understanding of RNAi sensitivity in this important insect order and identify critical future directions for research in this area, with an emphasis on using plant biotechnology approaches.

## Introduction

Of all arthropod orders, the insect order Coleoptera (beetles) contains the greatest number of described species (Stork, 2018). While many beetle species perform valuable agroecological services, such as biological control and pollination (Rader et al., 2016), others can be highly damaging pests of crops and forest trees. The most common solution to managing important coleopteran plant pests globally is the use of synthetic broad-spectrum insecticides. In addition, organic and other ecologically intensified farming systems use environmentally sound management techniques, such as enhancing conservation biological control, creating refuge areas, mixed cropping systems, and the use of cover crops. Plant protection biotechnologies are increasingly considered for combating coleopteran pest pressure, and such technologies include the use of *Bacillus thuringiensis* toxins and double-stranded RNA (dsRNA).

dsRNA currently represents the most species-specific approach to managing plant pests, given its nucleotide sequence-specific mode of action that relies on base-pairing between applied exogenous dsRNA (processed *in vivo* into segments 20–24 nucleotides in length) and complementary endogenous messenger RNA (mRNA). Generally, the complementarity of 21 consecutive nucleotides initiates the cleaving of the base-paired mRNA region, preventing translation of the targeted mRNA, a process known as RNA interference (RNAi). This unprecedented target-specificity is the basis for RNAi’s promise for biosafety to non-target organisms, and in turn the increasing interest in developing RNA-based biotechnology solutions to address plant protection. RNAi-based plant protection biotechnologies that have made significant progress include host-induced gene silencing (HIGS) technology *via* the use of transgenic cultivars containing RNAi traits, and spray-induced gene silencing (SIGS) technology *via* exogenous application of dsRNA to plants. Open-field RNAi-based management of western corn rootworm (*Diabrotica virgifera*) will commence in the United States in 2022 (followed by Canada in 2023), *via* the HIGS approach of consuming the tissues of the maize cultivar SmartStax PRO (Bayer, Germany), containing an RNAi-inducing trait. The biotechnology company GreenLight Biosciences (United States) is currently developing a SIGS approach against this pest Colorado potato beetle (*Leptinotarsa decemlineata*).

Investigations into RNAi-based management of coleopteran plant pests began with Baum et al. (2007), who demonstrated high dietary RNAi sensitivity (e.g., achieving target phenotype post-consumption of low amounts of dsRNA) in both *D. virgifera* and *L. decemlineata*. Since then, there has been an ongoing narrative, in RNAi-based insect management literature, that most coleopterans are sensitive to dietary RNAi. While this sensitivity has been clearly demonstrated in some coleopteran plant pests, many coleopteran species that have been examined show little to moderate sensitivity to dietary RNAi, and the vast majority of Order Coleoptera has not been examined. For the coleopteran species that have been examined, no overview of RNAi efficacy between and within coleopteran taxa has yet been presented to the scientific community.

Our timely review provides a balanced overview of dietary RNAi studies conducted on coleopteran species and discusses impediments to our knowledge regarding the overall prospects for RNAi-based biotechnology solution in the management of coleopteran plant pests. Given the upcoming introduction of insect RNAi technology to the American plant protection marketplace *via* the cultivation of transgenic maize containing an RNAi trait against *D. virgifera*, together with the role of coleopteran herbivores in causing significant damage globally to crops and forest trees, this represents a timely and critical re-assessment of our current state of knowledge. Our assessment is standardized to examine studies using non-formulated dsRNA (e.g., without the use of nanoparticles that enhance cellular uptake and systemic RNAi) to provide a reasonable comparison within and between coleopterans thus far studied. Biotechnologies considered in the present review include transgenic RNAi plants, “naked” dsRNA applications and bacterially expressed dsRNA.

## Observed RNAi Sensitivities in Coleoptera

### Family Chrysomelidae

Leaf beetles (Family Chrysomelidae) feed on a variety of plant tissues, and all chrysomelid species are fully herbivorous, many being serious crop pests. Several studies have shown that dietary RNAi sensitivity is quite high in the two chrysomelids *D. virgifera* (Baum et al., 2007; Bolognesi et al., 2012; Bachman et al., 2013; Miyata et al., 2014; Niu et al., 2017; Vélez et al., 2020) and *L. decemlineata* (Baum et al., 2007; Miguel and Scott, 2015; Cappelle et al., 2016; Spit et al., 2017; Máximo et al., 2020; Petek et al., 2020; Ren et al., 2021). Notably, Petek et al. (2020) demonstrated, in a small field study in Slovenia, that treating plants with water containing 0.01 μg dsRNA/μl significantly reduced *L. decemlineata* infestation in the field. This study was a landmark, as it suggests potential for dsRNA spray-based management of a major agricultural pest in open-field conditions, by mixing dsRNA with water alone.

Another chrysomelid, the mustard leaf beetle (*Phaedon cochleariae*), was recently examined for its potential as a screening model for coleopteran pests, and this species also demonstrated high RNAi sensitivity, with a strong lethal phenotype observed *via* consumption of leaf disks treated with water containing 0.025 μg dsRNA/μl (Mehlhorn et al., 2021). Zhang et al. (2019) demonstrated that painting a thin layer of water, containing a diluted suspension of dsRNA-expressing bacteria, on fresh willow leaves resulted in approximately 40 and 80% mortality of *Plagiodera versicolora* larvae after 2 and 7 days, respectively. More recently, Xu et al. (2021) painted poplar leaves with dsRNA to a concentration of 8 ng/cm^2^ and demonstrated 100% mortality in *P. versicolora* larvae within 5–7 days, depending on target gene. Notably, the authors observed over 60% mortality after 3 days of feeding on dsRNA targeting *actin* (see Supplementary Material in Xu et al., 2021). These studies with *P. versicolora*, together with that of Máximo et al. (2020), who observed effective *L. decemlineata* mortality after 2 days, suggest that RNAi can work in a timely manner in some coleopterans, perhaps in particular chrysomelids of the subfamily Chrysomelinae. Furthermore, studies performed in chrysomelids collectively suggest high dietary RNAi efficacy *via* various sources of dsRNA consumption (e.g., RNAi cultivar., naked dsRNA, and bacterially expressed dsRNA).

### Family Tenebrionidae

Darkling beetles (Family Tenebrionidae), while occupying a diverse array of ecological niches, consist of several important plant product pests, particularly in cereal and flour silos, as well as other stored food products. To our knowledge, the only tenebrionid species examined using dietary RNAi is the red flour beetle (*Tribolium castaneum*), a pest of global importance, and the existing data on dietary RNAi efficacy in *T. castaneum* are variable. Whyard et al. (2009) reported, after 7 days of dietary exposure, an LC_50_ of 0.0025 μg dsRNA/mg diet, and Laudani et al. (2017) reported approximately 30% mortality after 3 days of feeding on 0.015 μg dsRNA/mg diet, both studies being in contrast to Cao et al. (2018) who reported far more delayed RNAi in *T. castaneum via* feeding on 17- and 33-fold higher concentrations than those used by Laudani et al. (2017). Showing intermediate efficacy relative to the abovementioned studies, Abd El Halim et al. (2016) demonstrated 50% mortality after 6 days of feeding on 0.1 μg dsRNA/mg diet. As each study, with the exception of Abd El Halim et al. (2016), targeted *vATPase* components, these contrasting results clearly indicate a need for more investigations in order to solidify any conclusions regarding dietary RNAi efficacy in *T. castaneum*. Furthermore, expanding dietary RNAi studies to consider other important tenebrionid pests represent a critical route toward future biotechnological solutions to protect plant-based foods globally.

### Family Coccinellidae

Lady beetles (Family Coccinellidae) are an important group of coleopterans, regarding plant protection, due to different species’ roles as predators of crop pests (e.g., *Hippodamia convergens*, *Coleomegilla maculata*, and *Coccinella septempunctata*), invasive pests that outcompete beneficial lady beetles (e.g., *Harmonia axyridis*), and herbivorous pests that can be highly damaging to crop yields (e.g., *Henosepilachna vigintioctopunctata*). Targeting the same gene in four lady beetle species (*H. convergens*, *H. axyridis*, *C. maculata*, and *C. septempunctata*), Pan et al. (2020) observed approximately 80, 5, 60, and 70% mortality, respectively. These results indicate that dietary RNAi efficacy may be highly variable between coccinellid species. Furthermore, the concentration of dsRNA in the diet provided to coccinellids during the abovementioned study (4 μg dsRNA/μl) was rather high in relation to what would be realistically applicable in RNAi-based plant protection, suggesting the need to re-evaluate the dietary RNAi susceptibility of some of these more RNAi-sensitive species, using more economically feasible dsRNA concentrations.

Lü et al. (2020a,b, 2021a,b) and Guo et al. (2021) examined RNAi efficacy *via* oral delivery of dsRNAs targeting various genes in the 28-spotted potato ladybird (*H. vigintioctopunctata*). Lü et al. (2020b) observed approximately 60% mortality (8 days, target gene *Snf7*) and 70–85% mortality (10 days, targeting eight different coatomer components) *via* feeding on leaves treated with dsRNA at a concentration of 0.005 μg dsRNA/μl. Guo et al. (2021) observed nearly complete mortality after 7 days (target gene *vATPase E*) of feeding on leaves treated with dsRNA at a concentration of 0.01 μg dsRNA/μl. These studies by Lü et al. (2020a,b, 2021a,b) and Guo et al. (2021) additionally examined dietary RNAi efficacy in *H. vigintioctopunctata* first- and third-instar larvae and adults, using bacterially expressed dsRNA and demonstrated higher and faster RNAi efficacy in larvae compared to adults. Finally, Ren et al. (2021) observed significant growth retardation in *H. vigintioctopunctata via* consumption of transgenic potato plants. While sufficient data now exist for *H. vigintioctopunctata*, indicating its sensitivity to dietary RNAi, especially when fed leaves treated with low dsRNA concentrations (both naked and bacterially expressed), more data are required with regard to other coccinellid species, both pests and beneficials.

### Family Curculionidae

True weevils (Family Curculionidae) make up the largest coleopteran family. Many species are plant pests, and several have been studied with regard to their sensitivity to dietary RNAi. Christiaens et al. (2016) demonstrated, in the sweetpotato weevil *Cylas brunneus*, 46–49% mortality (depending on target gene) after 7 days of feeding on artificial diet containing dsRNA at a concentration of 0.01 μg dsRNA/μl. *C. brunneus* mortality reached 85–89% after 14 days. Prentice et al. (2019) demonstrated, in the sweetpotato weevil *Cylas puncticollis*, that 7 days of feeding on artificial diet treated with dsRNA (target gene *Snf7*) at a concentration of 0.01 μg dsRNA/μl resulted in 36% mortality after 14 days. Wu et al. (2019) observed no effect on survival of pepper weevil (*Anthonomus eugenii*) after dietary exposure to pepper coated with dsRNA (target genes *Snf7* and *vATPase A*) at a concentration of 0.3 μg dsRNA/μl. Pinheiro et al. (2020) observed between 43 and 93% mortality in the Sri Lanka weevil (*Myllocerus undecimpustulatus*), depending on the target gene. However, as mortality was only monitored after 26 days (dsRNA treatments were administered for 20 days), there remain critical unknowns with regard to timeliness of RNAi-induced mortality. Salvador et al. (2021) reported that 14 days of dietary exposure, using artificial diet overlaid with treatment solutions containing 0.2 μg dsRNA/μl, resulted in 40–60% mortality in cotton boll weevil (*Anthonomus grandis*) larvae, and 10–30% mortality in adults, percent mortality depending on target gene in both life stages.

Finally, Kyre et al. (2019) provided a sucrose-based liquid diet containing 10 μg dsRNA/μl to southern pine beetle (*Dendroctonus frontalis*) adults. While this dsRNA concentration is unrealistically high in relation to what is feasible for RNAi-based plant protection, they nevertheless provided a proof-of-concept that this forest pest species is susceptible to dietary RNAi. Here, RNAi-induced mortality ranged from completely ineffective to 100% mortality, depending on target gene. Taking these data on *D. frontalis*, and further examining this species’ RNAi sensitivity when exposed to field-realistic concentrations of dietary dsRNA, represents a vital step toward understanding the potential for controlling this forest pest. More broadly, curculionids clearly show variable susceptibility to dietary RNAi. Curculionidae being the largest family in Order Coleoptera, with many curculionid species being detrimental to economically important plant species globally, much more research is needed in order to conclude any patterns regarding RNAi sensitivity in this family. Further studies with curculionids would benefit from including multiple sources of dsRNA consumption (e.g., RNAi cultivar., bacterially expressed dsRNA), as thus far studies in weevils have only examined naked dsRNA in artificial diet.

### Family Nitidulidae

Sap beetles (Family Nitidulidae) represent an important family containing several very important plant pest species. The first nitidulid to be examined for sensitivity to dietary RNAi was the small hive beetle (*Aethina tumida*), a facilitative pest of flowering plant species due to its status as a major pest of honey bees (honey bees being important for sexual reproduction of many flowering plant species). Powell et al. (2017) reported 73–93% adult *A. tumida* emergence (depending on target gene), compared 100% adult emergence in control group, after 6 days of dietary exposure to 33 μg dsRNA/g artificial diet.

The only other nitidulid to be examined thus far *via* dietary RNAi is the pollen beetle *Brassicogethes aeneus*. Knorr et al. (2018) observed at least 90% adult *B. aeneus* mortality after 14 days, in all four target gene treatments examined, after dietary exposure to water containing 1 μg dsRNA/μl followed by continuous uptake of dsRNA in artificial diet containing 500 ng/cm^2^. Willow et al. (2020, 2021a,b,c) examined RNAi sensitivity in *B. aeneus via* multiple field-relevant routes of exposure, and in both adults and larvae. A SIGS approach to achieving RNAi-induced mortality in *B. aeneus*, *via* feeding on dsRNA-treated oilseed rape buds and anthers, leads to variable success in achieving a lethal phenotype in this pest species. Most notably, high concentrations of 2.5–5 μg dsRNA/μl proved effective after 3 days of feeding on dsRNA-treated anthers, while these concentrations and a lower concentration of 0.5 μg dsRNA/μl showed significantly greater efficacy when dsRNA-treated anthers were provided for 17 days (Willow et al., 2021b). This is an important finding due to constant development and senescence of oilseed rape flowers, making successive sprays over the growing season a potential necessity for implementing a SIGS approach. In contrast, 3 days of feeding on treated oilseed rape buds, even when using a dsRNA concentration as high as 5 μg dsRNA/μl, showed negligible effect on *B. aeneus* adult survival (Willow et al., 2020).

The abovementioned studies indicate that both *A. tumida* and *B. aeneus* demonstrate little to moderate susceptibility to dietary RNAi. Further studies examining field-relevant dsRNA concentrations in a SIGS approach may provide evidence necessary to make conclusions regarding RNAi susceptibility in these two important pest species. The significantly enhanced RNAi efficacy from chronic dsRNA feeding, in *B. aeneus* (Willow et al., 2021b), also suggests potentially important benefits of developing RNAi cultivars to managing *B. aeneus* or other pests showing similar differences in RNAi efficacy between short-term and chronic dsRNA feeding. Furthermore, other economically important nitidulids (e.g., *Stelidota geminata* and *Carpophilus* spp.) should be examined, in order for us to understand more about the potential for implementing RNAi to manage nitidulid pests.

### Families Buprestidae and Cerambycidae

Jewel beetles (Family Buprestidae) and longhorn beetles (Family Cerambycidae) consist of herbivorous species that feed on the tissues on roots, stems, and leaves of various herbaceous and woody plants. Some species are wood-borers that are highly damaging to forest trees. The only buprestid to be examined for dietary RNAi susceptibility is emerald ash borer (*Agrilus planipennis*). Rodrigues et al. (2017) exposed *A. planipennis* larvae to dietary dsRNA (*in vitro* synthesized) in sucrose solution for 4 days, and mortality of *A. planipennis* was monitored at 10 days. The authors observed 30, 35, and 78% larval mortality associated with 1, 6, and 10 μg dsRNA/μl treatments, respectively. More recently, Leelesh and Rieske (2020) demonstrated 47–69% mortality (depending on target gene) in *A. planipennis* larvae fed bacterially expressed dsRNA (at approximately 100 bacterial cells/nl).

The only cerambycid to be examined for dietary RNAi susceptibility is Asain long-horned beetle (*Anoplophora glabripennis*). Dhandapani et al. (2020a) exposed *A. glabripennis* larvae to dietary dsRNA (*in vitro* synthesized) in sucrose solution for 3 days, and mortality was monitored up to 10 days. The authors observed (depending on target gene) 17–25%, 50–67%, and 75–90% larval mortality associated with force-fed doses of 2, 5, and 10 μg dsRNA/day, respectively. Another study reported approximately 20% mortality, after 20 days, in both larval and adult *A. glabripennis* that were fed 15 μg dsRNA/day for 3 days (see Supplementary Material in Dhandapani et al., 2020b).

As the concentrations/doses used in the abovementioned buprestid and cerambycid studies are all above what is realistic for pest management, together with the fact that these wood-boring species would not be exposed to dsRNA through sucrose solutions or dsRNA-expressing bacteria, more studies should be conducted using field-realistic concentrations, and *via* field-relevant exposure routes (e.g., vascular cambium and wood). Furthermore, studies examining dietary RNAi susceptibility in wood-boring beetles may benefit greatly from the development of RNAi cultivars of the relevant tree species, for use in experiments; this is important, as a HIGS approach may represent the most economic and effective RNAi technique for managing forest tree pests.

## Conclusion and Future Directions

Few families in the order Coleoptera have been examined for dietary RNAi efficacy. Furthermore, within each family examined, few species are represented in the existing dietary RNAi literature. Surprisingly, our findings from this review indicate that less than half of the coleopteran species thus far examined for dietary RNAi efficacy are sensitive to dietary RNAi. While methods and concentrations used are variable within chrysomelid studies, all chrysomelid species thus far examined seem to show high sensitivity to dietary RNAi, a possible reason for the ongoing narrative that most coleopterans have high sensitivity to dietary RNAi. The species considered here to be insensitive to dietary RNAi have either demonstrated very little RNAi-induced mortality, or effective lethality of dsRNA has been observed for these species only when using unrealistically high concentrations of dsRNA. Considering that these studies are conducted after dsRNA-microinjection bioassays have already taken place, indicating what would theoretically be the most appropriate gene to target for inducing a lethal phenotype upon dsRNA ingestion, the dietary RNAi-insensitivity of more than half of all coleopterans examined is alarming. As some studies have indicated the role of dsRNases in lowering, limiting, or preventing dietary RNAi in certain coleopteran species (Almeida Garcia et al., 2017; Spit et al., 2017; Prentice et al., 2019; Peng et al., 2020), this potential limiting factor should be investigated in other coleopteran species for which dietary RNAi sensitivity is observed to be low. Furthermore, numerous studies have only examined routes of exposure that are not applicable to real-world pest management.

Source of dsRNA consumption represents an important variable when determining dietary RNAi sensitivity in any organism. Other than the three dsRNA-sensitive species *D. virgifera* (Baum et al., 2007; Vélez et al., 2020), *L. decemilineata* (Ren et al., 2021), and *H. vigintioctopunctata* (Ren et al., 2021), no other coleopteran has been examined for RNAi cultivar-based management efficacy. Such studies would contribute much to forwarding our knowledge regarding the prospects for using transgenic approaches to RNAi-based management of coleopteran plant pests. Most studies in coleopterans examine potential for RNAi-based management *via* the use of naked dsRNA in artificial diets. This has allowed us to make general comparisons, regarding dietary RNAi efficacy, across and between taxa. The use of naked dsRNA also enables accurate reporting of dsRNA doses/concentrations examined. Some studies reported in our review examine dietary RNAi efficacy *via* the use of bacterially expressed dsRNA. In the case of the lady beetle *H. vigintioctopunctata*, dietary RNAi efficacies are similar whether using naked dsRNA or bacterially expressed dsRNA.

Other than the need to examine a greater diversity of pest species within the coleopteran families already discussed in the present review, there are numerous other important coleopteran taxa deserving of attention with regard to potential susceptibility to dietary RNAi. These include not only pest species, but also species that are important for directly providing ecosystem services (e.g., biological control, soil decomposition, and pollination), or species that play more facilitative roles in maintaining ecosystem biodiversity (e.g., those which are important food sources for higher trophic levels). Understanding dietary RNAi sensitivity in these beneficial coleopterans should be of great interest due to the concern for safeguarding non-target organisms. Furthermore, as co-formulating dsRNA with various types of nanoparticles can enhance dsRNA stability and RNAi efficacy in various insects (Pugsley et al., 2021), thereby making such formulations vital for SIGS approaches to plant protection, the use of different dsRNA−nanoparticle complexes continues to represent a critical avenue of investigation in both pest and beneficial coleopterans.

Finally, dsRNA resistance has been demonstrated *in vivo via* selective breeding in both *D. virgifera* (Khajuria et al., 2018) and *L. decemlineata* (Mishra et al., 2021), which is of great concern RNAi-based management of coleopteran pests. Yoon et al. (2018) observed that the dsRNA binding protein Staufen isoform C (*stauC*) is present in 24 of the 32 beetle genomes/transcriptomes they analyzed. The authors subsequently demonstrated *stauC* mRNA was significantly less expressed in an RNAi-resistant *L. decemlineata* cell line, and that in these cells, processing of dsRNA into small interfering RNAs was reduced by more than 80% compared to a susceptible *L. decemlineata* cell line. While the abovementioned study also suggests *stauC* downregulation as a catalyst for RNAi resistance in *D. virgifera* and *T. castaneum*, *stauC*’s presence in only three-fourths of the coleopteran species bioinformatically analyzed in the study suggests a diversity of RNAi resistance potential and/or mechanisms within Coleoptera. RNAi being a promising and biosafe technology for plant protection, these findings on RNAi resistance support the need for investigations into precise RNAi resistance mechanisms among different coleopteran taxa.

RNAi technology will likely play a major role in plant protection against coleopteran insects, given the current momentum in both research and development for the use of this biotechnology to protect both crop yields and forest trees. For RNAi-sensitive chrysomelid species, both HIGS and SIGS approaches are in development for the plant biotechnology marketplace. HIGS, through the development and use of RNAi cultivars that constantly produce target-specific dsRNA, may be the most appropriate method of dsRNA delivery when dealing with plant pests that only show effective dietary RNAi when chronically exposed to dsRNA. This transgenic approach is also likely an appropriate direction for managing tree pests, as successful RNAi-based management of these pests seems more likely if the target-specific dsRNA is constantly produced in the tree tissues, as opposed to forest management’s reliance on a future of accurately timed trunk injections. That being said, it remains critical to examine possibilities for using both transgenic and non-transgenic approaches to managing coleopteran plant pest species.

## Author Contributions

JW conceived the manuscript and wrote the original draft. All authors made comments or suggestions toward revising the original draft and approved the final version of the manuscript.

## Funding

The review was funded by the Personal Research Funding project no. PRG1056 of the Estonian Research Council.

## Conflict of Interest

The authors declare that the research was conducted in the absence of any commercial or financial relationships that could be construed as a potential conflict of interest.

## Publisher’s Note

All claims expressed in this article are solely those of the authors and do not necessarily represent those of their affiliated organizations, or those of the publisher, the editors and the reviewers. Any product that may be evaluated in this article, or claim that may be made by its manufacturer, is not guaranteed or endorsed by the publisher.
